# Characterization of the Occult Hepatitis B Virus Variants Circulating among the Blood Donors from Eastern India

**DOI:** 10.1155/2013/212704

**Published:** 2013-11-04

**Authors:** Avik Biswas, Rajesh Panigrahi, Partha Kumar Chandra, Arup Banerjee, Sibnarayan Datta, Manisha Pal, Subhashish Chakraborty, Prasun Bhattacharya, Sekhar Chakrabarti, Runu Chakravarty

**Affiliations:** ^1^ICMR Virus Unit, Kolkata, GB 4, 1st Floor, ID & BG Hospital Campus, 57 Dr. Suresh Chandra Banerjee Road, Kolkata 700010, India; ^2^Tulane University Health Sciences Center, Department of Pathology and Laboratory Medicine, 1430 Tulane Avenue SL-79, New Orleans, LA 70112-2699, USA; ^3^Translational Health Science and Technology Institute (THSTI), Gurgaon, Haryana 122016, India; ^4^Defence Research Laboratory, Tezpur, Assam 784001, India; ^5^Department of Statistics, University of Calcutta, Kolkata 700019, India; ^6^Institute of Blood Transfusion Medicine and Immunohematology, Kolkata 700006, India; ^7^Medical College & Hospital, Kolkata 700073, West Bengal, India; ^8^National Institute of Cholera and Enteric Diseases, Kolkata 700010, India

## Abstract

A previous study from West Bengal documented very high rate of occult HBV infection (OBI) among the HBsAg negative blood donors. This study was aimed to characterize the OBI strains circulating among the blood donors and to estimate the risk associated with the prevailing viral variants/mutants. Blood samples from 2195 voluntary blood donors were included in the study. HBsAg, HBeAg, anti-HBc, and anti-HBs statuses of the samples were done by ELISA based detection. PCR amplification and sequencing were done to determine HBV genotypes, basal core promoter (BCP), and precore (Pre-C) mutations. Among the study samples, 268 were anti-HBc positive/HBsAg negative, among which 65 (24.25%) were HBV DNA positive. Phylogenetic analysis revealed the presence of HBV/D (87.23%), HBV/A (8.51%), and HBV/C (4.26%) (*P* < 0.0001). HBV/D3 (65.85%) was the significantly prevalent subgenotype over HBV/D2 (26.83%) and HBV/D1 (7.31%) (*P* = 0.0003). Considerable prevalence of differential BCP (1752C, 1753C, 1762T/1764A, 1753C+1762T/1764A, 1773C, and 1814C) and reverse transcriptase (rt) gene (rtI91L, rtL93P, rtS106C, rtR110G, rtN118T, rtS119T, rtY126H, rtG127W/R, rtC136R, and rtY158H) mutations was identified. Association of specific HBV subgenotypes with OBI was interesting and needs further study. Clinically relevant mutations were prevalent among the OBI strains which are of serious concern.

## 1. Introduction

The serological diagnosis of hepatitis B virus (HBV) infection is mainly based on hepatitis B surface antigen (HBsAg) detection assays, and the absence of HBsAg is believed to exclude infectivity. However, presence of HBV DNA in circulation/liver without detectable HBsAg, with or without the presence of any other HBV antibodies, is defined as occult HBV infection (OBI) [1]. 

The exact mechanism of OBI is still a matter of debate. Several different mechanisms are reported to be associated with OBI which includes mutations in S gene (specifically mutations in the major hydrophilic loop or in the “a” determinant region, which is the main target for antibodies used in diagnostic tests) that may alter HBsAg structure and expression; decrease of HBV replication and/or hindrance of HBsAg detection. However, some studies also showed that majority of occult HBV infections are caused by wild-type HBV strains [2]. 

So far, the clinical significance of OBI is largely unknown. Detection of OBI has been reported in subjects with clinical manifestations, such as chronic liver disease and hepatocellular carcinoma (HCC) [3]. Although most of the OBI carriers are asymptomatic, OBI has been detected in patients with cryptogenic chronic liver disease [4, 5] and could be associated with progression of liver fibrosis and cirrhosis development [3]. 

So far, up to ten genotypes (A through J) and several subgenotypes are identified and characterized [6–10], which show distinct geographical distribution pattern. There is growing evidence that genotypes influence the severity of liver diseases and also response to antiviral treatment [7, 11–13]. 

HBV mutant strains with mutations in the basal core region (BCP) and precore (pre-C) regions are studied, among which BCP double mutation (1762T/1764A) and pre-C stop codon mutations (1896A) are important. Other mutations in the BCP and pre-C region include 1752C, 1753C, 1757A, 1766T/1768A double mutation, 1773C, 1814C/T, 1858C, 1862T, 1888A, and 1899A. BCP double mutations are less frequent in asymptomatic carriers but are frequently detected in patients with HCC [14]. Additionally several studies reported mutations in the HBV reverse transcriptase (rt) domain leading to treatment failure [15, 16]. A recent study documented the existence of HBVrt mutations among the chronic carriers of Eastern India [17].

So far, very few studies characterized the viral strains and viral genotypes circulating among the OBI positive individuals. A previous study from Eastern India reported very high rate of OBI among the voluntary blood donors [1]. But data regarding the frequency of clinically relevant BCP/pre-C mutation and the rate of differential drug resistant HBVrt mutations are not there. Thus, this study was aimed to characterize the OBI strains circulating among the voluntary blood donors to determine the significance and the degree of risk factors associated with the different viral variants/mutants. 

## 2. Materials and Methods

### 2.1. Study Subjects

A total of 2195 blood samples collected from the voluntary blood donors during the time period June 2006 to May 2009 were included in the study. Up to 3.0 mL of blood was collected, from which plasma/serum was separated and stored at −80°C until further used. The study includes two projects approved by the respective institutional ethical committees. The informed signed consent of each donor prior to blood donation was also collected. 

### 2.2. Serological Assays

HBsAg (HBsAg Uni-Form II; Hepanostika, Boxtel, The Netherlands), anti-HBc antibody (anti-HBc total, i.e., both for IgM and IgG) (Hepanostika anti-HBc Uni-form test, Biomerieux, The Netherlands), HBeAg (EQUIPAR, Varese, Italy), and the presence or absence of anti-HBs antibodies (Anti-HBs EIA, Diasorin, Saluggia, Italy) were determined. 

After initial collection, all the samples were tested for the presence of anti-HBc antibody. Then, anti-HBc positive samples (after testing positive twice) were tested for HBsAg by ELISA. Anti-HBc(+ve)/HBsAg(−ve) samples were further tested for the presence of anti-HBs antibodies. All kinds of ELISA testing were done twice to confirm the findings.

### 2.3. Extraction of DNA from Plasma/Serum

From the anti-HBc(+ve)/HBsAg(−ve) samples, DNA was extracted from 200 *µ*L of serum by using QIAamp DNA blood Mini Kit (Qiagen, Hilden, Germany). The final elution volume was 200 *µ*L.

### 2.4. HBV DNA Detection, Quantification, PCR Amplification of Surface Gene Region, and Basal Core Promoter/Precore Region

Detection of the presence of HBV DNA was done by sensitive nested PCR amplification of HBV small surface gene region [18]. Additionally, the HBV surface gene PCR amplified samples were further amplified for BCP/pre-C gene region [19, 20]. Precautions were taken during amplification process to protect against carry over contamination and false positive HBV DNA on PCR [21]. In addition, each sample was tested in duplicates, and negative controls were included during each assay. HBV DNA quantification was done by real time PCR detection assay as previously described [18].

### 2.5. HBV Genotyping/Subgenotyping and Cloning

PCR products are directly sequenced in both directions as previously described [22]. After sequencing, sequences were aligned by ClustalW multiple alignment programme, compared, and manually edited using BioEdit v7.0.4.1 program [23]. HBV genotypes and subgenotypes were assigned by phylogenetic analysis of surface gene region as previously described [22]. To verify the presence of minor subpopulations of virus pool, PCR product cloning was done as described previously [22]. 

The nucleotide sequences used in this study have been submitted to the DDBJ/EMBL/GenBank, accession numbers JQ388839–JQ388883.

### 2.6. Statistical Analysis

Statistical analysis of the data has been performed using MINITAB Statistical Software (version: 13.31, Minitab Inc., PA, USA). Frequency *χ* ^2^-test has been used to examine association between two variables. Proportions have been tested wherever necessary. A *P* value less than 0.05 has been considered as statistically significant. 

## 3. Results

### 3.1. Baseline Characteristics of the Study Subjects

Among the 2195 collected blood samples, 268 were anti-HBc positive/HBsAg negative. Among these 268 anti-HBc positive/HBsAg negative cases 65, were positive for HBV DNA; thus, the rate of OBI among the anti-HBc positive population was 24.25% (65/268), and the overall OBI rate was 2.96% (65/2195).

The demographic, serological, and virological characteristics of the 65 occult HBV infected blood donors are presented in the Table 1. 

 All the 268 anti-HBc positive/HBsAg negative samples were found to be negative for HBeAg serology.

### 3.2. HBV Genotype and Subgenotype Distribution

**Figure 1:**
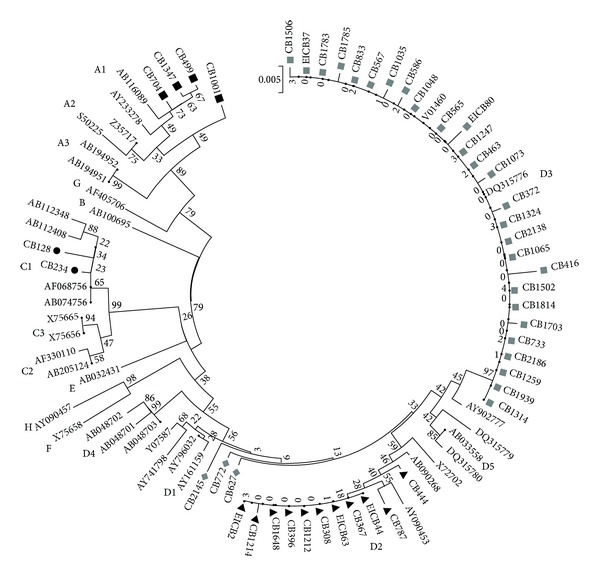
Phylogenetic analysis of partial S gene region sequence (300 base pairs) of HBV isolates from Eastern Indian voluntary blood donors with OBI (denoted with “CB”). Different genotypes and subgenotypes are assigned with respect to reference sequences retrieved from the GenBank. The phylogenetic tree was constructed by neighbor joining (NJ) method. The bootstrap values obtained from 1,000 replicates are given at the internal nodes.

**Figure 2:**
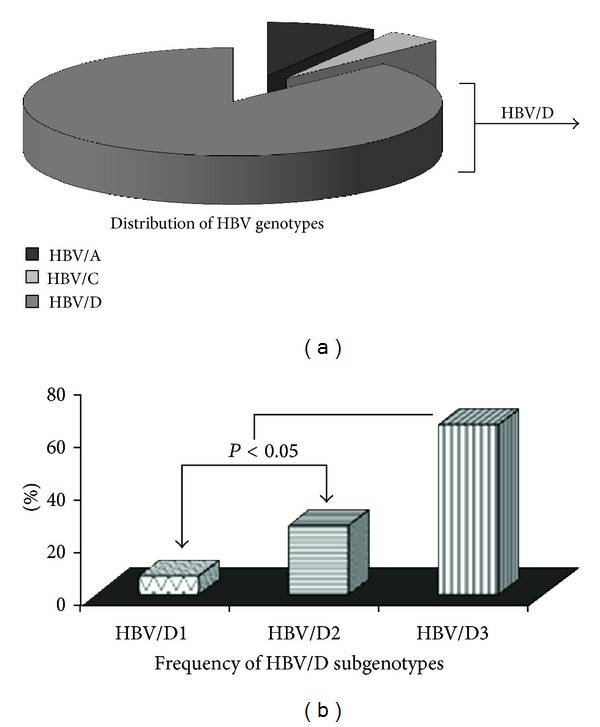
Distribution of HBV genotypes/subgenotypes among the blood donors is represented by pie diagram. The frequencies of different subgenotypes of HBV/D are also depicted. The *P* value (<0.05) indicates that the prevalence of HBV/D3 was significantly higher in comparison to HBV/D1 and HBV/D2.

Initially, all the 65 HBV DNA positive samples were targeted for sequencing analysis, but only 47 (47/65; 72.31%) samples could be successfully sequenced for HBV partial surface gene region and analyzed for genotyping. Phylogenetic analysis of the sequences showed that three HBV genotypes are prevalent (namely, HBV/A, HBV/C, and HBV/D) (Figure 1). Overall, HBV/D was found to be the significantly predominant strain (41/47; 87.23%), followed by HBV/A (4/47; 8.51%) and HBV/C (2/47; 4.26%) (*P* < 0.0001) (Table 1 and Figure 2). For HBV/A and HBV/C, only one subgenotype could be identified (HBV/A1 (Aa) and HBV/C1 (Cs), resp.). Among the HBV/D genotypes, three subgenotypes were prevalent, namely, HBV/D1 (3/41; 7.31%), HBV/D2 (11/41; 26.83%), and HBV/D3 (27/41; 65.85%). Among the different HBV subgenotypes identified, HBV/D3 was the significantly prevalent strain over HBV/D1 and HBV/D2 (*P* = 0.0003) (Figure 2). 

### 3.3. Prevalence of HBV Subtypes/Serotypes

Overall, 5 different subtypes could be identified, namely, “adw2,” “adr,” “adw3,” “ayw2,” and “ayw3.” All the 4 HBV/A strains were found to be of subtype “adw2,” and the 2 HBV/C strains were of “adr” subtype. All 3 HBV/D1 strains were of “ayw2” serotype, while 10 HBV/D2 strains were of “ayw3” serotype and 1 of “adw3” serotype. In case of all the 27 HBV/D3 isolates, “ayw3” was found to be the only HBV serotype. 

### 3.4. Analysis of Surface Gene Region

Sequence analysis of the “a” determinant region (amino acid 96–160) of the surface gene revealed that multiple amino acid (aa) substitutions/mutations exist among the OBI cases. At least 4 different genotype/subgenotype unrelated aa mutations are observed: V96A (1/47; 2.13%), L98V (1/47; 2.13%), Y100C (1/47; 2.13%), and I1110L (3/47; 6.38%) (data not shown). 

### 3.5. Analysis of Basal Core Promoter (BCP) and Precore (Pre-C) Region

BCP region could be successfully amplified and sequenced for 27 samples (which includes HBV/A = 4, HBV/C = 1, HBV/D1 = 2, HBV/D2 = 8, and HBV/D3 = 12). Overall, multiple BCP and pre-C mutations were found with differential frequency; 1752C (4/27; 14.81%), only 1753C (4/27; 14.81%), 1753C+1762T/1764A (2/27; 7.41%), 1762T/1764A (2/27; 7.41%), 1773C (11/27; 40.74%), 1814C (7/27; 25.93%), 1858C (10/27; 37.04), and 1862T (7/27; 25.93). The frequencies of the BCP and pre-C mutations among the different HBV subgenotypes are shown in Table 2. In case of one HBV/D3 isolate, a small 5 bp deletion (nt 1834–1838) was found. 

In none of the isolates, 1766T/1768A double mutation in BCP region and 1896A/1899A mutations in pre-C region could be detected.

### 3.6. Analysis of Polymerase Gene Region

The HBV polymerase gene reverse transcriptase (rt) region has been divided into six distinct domains (F and A–E). Among these six domains, 5 interdomain regions exist. Among the studied samples, domain A and B were found to be conserved across HBV genotypes and subgenotypes. The variability was observed in the A-B interdomain region. Multiple mutations were found in the A-B interdomain region, such as rtI91L, rtL93P, rtS106C, rtR110G, rtN118T, rtS119T, rtY126H, rtG127W/R, rtC136R, and rtY158H (data not shown). Some novel (rtS119T, rtY126H, rtG127W/R, rtC136R, and rtY158H) and nonclassical putative (rtI91L) mutations were found. 

During analysis, amino acid positions were considered according to the reverse transcriptase domain start site that is, highly conserved “EDWGPCDEHG…” motif  [24]. 

### 3.7. HBV Quasispecies Analysis

HBV quasispecies study by cloning analysis of the HBV surface gene region was done for 11 randomly chosen OBI isolates. For each isolate, at least 12 clones were amplified, of which at least 10 amplified products were sequenced. During quasispecies analysis, 300 bp partial surface gene sequences were analyzed. Of these 11 isolates, different HBV subgenotypes were included for analysis (HBV/A = 2; HBV/C1 = 1; HBV/D1 = 1; HBV/D2 = 4, and HBV/D3 = 3). 

In none of the isolates, any other minor subpopulation of genotype/subgenotype could be identified. In 3 isolates (3/11; 27.27%) (2 HBV/D2 and 1 HBV/D1), presence of HBV quasispecies was found. The percent nucleotide identity among the subpopulations of these three isolates was 99.6 ± 0.31; 99.86 ± 0.19; and 99.82 ± 0.22, respectively. Of these 3 isolates, 2 were found to have nonsynonymous nucleotide changes at the quasispecies level. In one case, A159G and R160K amino acid changes were found, and in the other, I68T amino acid substitution was observed. Notably, none of these three isolates with the presence of HBV quasispecies were anti-HBs positive.

## 4. Discussion

Transfusion transmitted HBV (TTHBV) still continues to be a major problem in India, despite the availability of the ELISA based HBsAg screening as the only mandatory test for detection of HBV infection in blood units before donations over the last few decades [25]. A previous study reported very high rate of OBI among the voluntary blood donors from Eastern India [1]. In the present study, molecular genetic characterization of the circulating OBI strains revealed some important facets of HBV infection that are expected to be important from the view point of molecular surveillance. Additionally, this information is valuable for the assessment of clinical potential of these strains.

 In this study, viral characterization revealed that HBV/D (87.23%) was the major strain, while HBV/A (8.51%) and HBV/C (4.26%) were detected in low proportions (Figure 2). HBV/A is reported to be associated with advanced liver disease in the same population [26]; thus, its lower prevalence in the asymptomatic blood donor study population was expected. A previous study reported the predominance of HBV/D among the anti-HBc only subjects [27]. Previous studies showed that in eastern India HBV/D was associated with asymptomatic/inactive carrier stage [26, 28] and HBV/D3 with HBsAg negative carrier state [19, 29]. HBV/D1 was reported to be associated with HBsAg positive infections [19], thus the lower frequency of HBV/D1 among the study subjects was not unusual. Previous studies from the same population reported the presence of HBV/D5 at a considerable level [19]; thus, its absence in the present study population was unusual. In the current study, all the HBV genotypes/subgenotypes are found to be restricted at the level of HBV serotypes. All the HBV/D3 and HBV/D2 (excepting a single isolate of HBV/D2) are of serotype “ayw3,” while HBV/A and HBV/C were of serotype “adw2” and “adr,” respectively.

Interestingly, no particular well known surface gene mutation that can affect the HBsAg detection by ELISA could be detected among the occult HBV strains. Mutations related to diagnostic failure (such as M133T/I, F134N, and S143L) including vaccine escape mutations such as D144A and G145R could not be found. Thus, no particular HBsAg structure disrupting mutation related with HBsAg detection failure could be identified. Notably, T125M substitution was exclusive among the HBV/D3 isolates (data not shown). Similar association of this T125M substitution with HBV/D3 strains was documented in previous studies [18, 29]. When the HBsAg overlapping HBV polymerase region was analyzed, considerable variability was observed in the HBV reverse transcriptase A-B interdomain region. In a previous study, we found considerable prevalence of putative mutations among the therapy naïve seropositive patients [17]. The clinical and therapeutic significance of the mutations in the HBVrt region among the asymptomatic carriers who are at occult stages of infection need further study. 

The clinical impact of OBI is still unclear. In this study, all the OBI subjects are apparently healthy. Thus, for the blood donors who are at the occult stages of infection but are otherwise healthy, HBV associated disease prognosis and transfusion associated HBV transmission are of concern. To assess the clinical consequences of these OBI positive blood donors, prospective longitudinal follow-up studies are needed in future. It has been suggested that immunologically competent individuals with OBI and no other concomitant liver disease generally do not show clinical evidence of liver damage [30]. The average ALT level of the OBI positive cases was 34.67 ± 8.14 IU/L; thus, no apparent sign of active liver disease was observed. Still, considering the fact that HBV associated disease prognosis is dependent on time and as well as on multiple factors, chances for development of liver complications in future exist. To add to the concern, different clinically relevant mutations in the BCP and polymerase gene region are found among the OBI blood donors in considerable proportions. In the BCP region mutations such as 1752C, only 1753C, 1753C + 1762T/1764A (triple mutation), and 1762T/1764A (double mutation) are noteworthy, and in HBVrt region, rtS106C mutation was found among the OBI isolates. A recent study from South India reported the presence of rtS106C mutation among the chronic HBV carriers [31]. Among the HBV/D infected Mongolian population, 1752C and/or 1753V (V = non T) mutation was reported to be associated with HCC [32]. Another study showed that 1753V mutations in addition to 1762T/1764A are associated with HCC among the HBV/C infected cases [33]. Several studies also suggested the role of mutations at the EnhII/BCP region (1753V and 1762T/1764A) with an increased risk of advanced liver diseases [34–36]. Additionally, a recent study from Eastern India showed that among the HBsAg positive subjects 1753C was common among cirrhosis cases and 1762T/1764A was frequent among chronic liver disease cases and are found to be critical factors for clinical advancement [26]. Thus, the presence of these BCP mutations (1752C, 1753C, 1753C+1762T/1764A, and 1762T/1764A) among the OBI blood donors in the same population is noteworthy. Hence, in the near future, follow-up studies are needed to assess the clinical relevance of these molecular variants among the OBI infected blood donors, as presence of OBI is supposed to be a risk factor for the development of cirrhosis and HCC [3, 37]. 

In case of HBV, presence of HBV quasispecies is a common phenomenon. In the study samples, 27.27% (3 out of 11) of the isolates are found to contain HBV quasispecies. Quasispecies diversity within the OBI samples was limited, which was evident by the high level percent nucleotide identity (99.6 ± 0.31; 99.86 ± 0.19 and 99.82 ± 0.22). Lower quasispecies diversity reflects low level of viral replication rate [38], and this observation was supported by the lower viral load of the OBI samples of this study. Interestingly, in one isolate, a quasispecies clone showed R160K substitution that resulted in serotype conversion, and thus, a unique mixed serotype pattern of infection was documented. In none of the isolates, any immune escape or vaccine escape quasispecies variety could be identified.

One limitation of the current study is the inability of the amplification of all the OBI positive samples for two different HBV genomic regions (HBV surface gene region and BCP/pre-C gene region). Some previous studies indicated that due to the structural complexity of the HBV genome, different genetic regions are not amplified with same efficiency in PCR assays [39–42]. 

Thus, this study highlighted the association of HBV subgenotype D3 with OBI and needs further study to characterize this unique association. The HBV carriers (OBI) bearing clinically relevant BCP and HBVrt mutations need regular follow-up to assess the clinical potential of these strains. Hopefully, these data will certainly help to develop better HBV management strategies in India.

## Figures and Tables

**Table 1:** Demographic, serological, and virological characteristics of the 65 occult HBV positive blood donors.

Characteristics	HBsAg (−)/antiH-Bc (+) (*n* = 268)
HBV DNA positive (%)	65 (65/268; 24.25%)
Age in years* (Mean ± S.D.)	33.92 ± 10.46
ALT* (IU/L)	34.67 ± 8.14
HBV viral load* (log_10_⁡copies/mL)	3.78 ± 0.80

HBV genotype by sequencing (%)	47 (47/65; 72.31%)
HBV/A	4 (4/47; 8.51%)^a^
HBV/C	2 (2/47; 4.26%)^a^
HBV/D	41 (41/47; 87.23%)^a^
D1	3 (3/41; 7.31%)^b^
D2	11 (11/41; 26.83%)^b^
D3	27 (27/41; 65.85%)^b^

*Mean ± Standard Deviation.

*P* ^a^ < 0.0001; *P* ^b^ = 0.0003.

**Table 2:** Frequency of basal core promoter and precore mutations among the occult HBV isolates.

Mutations (%)	HBV genotypes/subgenotypes				
	HBV/A1 (*n* = 4)	HBV/C1 (*n* = 1)	HBV/D1 (*n* = 2)	HBV/D2 (*n* = 8)	HBV/D3 (*n* = 12)
1752C	1 (25.0)	—	—	2 (25.0)	1 (8.3)
1753C	—	1 (100.0)	—	1 (12.5)	2 (16.6)
1762T/1764A	—	—	1 (50.0)	—	1 (8.3)
1753C + 1762T/1764A	1 (25.0)	—	—	1 (12.5)	—
1773C	1 (25.0)	1 (100.0)	—	5 (62.5)	4 (33.3)
1814C	1 (25.0)	—	—	—	6 (50.0)
1858C	3 (75.0)	—	—	1 (12.5)	6 (50.0)
1862T	2 (50.0)	—	—	—	5 (41.7)

Notably, 1766T/1768A mutation in BCP region and 1896A or 1899A mutations in pre-C region could not be detected.
